# Extra-axial chordoma of the distal femur: a clinical and radiological challenge—case report and literature review -

**DOI:** 10.1007/s00256-025-05041-x

**Published:** 2025-09-29

**Authors:** Lotje A. Hoogervorst, Thomas W. Borsje, Danique L. M. van Broekhoven, Bart Kaptein, Ana Navas Cañete, Shaho Hasan, Robert J. P. van der Wal

**Affiliations:** 1 Department of Orthopaedics, Leiden University Medical Center, Leiden, 2333ZA the Netherlands; 2 Department of Radiology, Leiden University Medical Center, Leiden, 2333ZA the Netherlands

**Keywords:** Chordoma, Extra-axial, Oncology, Bone tumor, Orthopedics, Surgery

## Abstract

We report a very rare case of an intraossesous extra-axial chordoma (EAC) involving the distal femur. We describe the clinical presentation, radiologic and pathological findings, and the treatment. In addition, a systematic literature search was performed to further expand the knowledge regarding EAC in the lower extremities, including 13 papers describing a total of 21 adult patients.

## Introduction

Chordomas are rare malignant bone tumors, with an estimated incidence of 0.08–0.5 per 100,000 persons worldwide [1–5]. As chordomas develop from cellular remnants of the notochord, they typically occur at the skull base, the craniocervical junction, the mobile spine, and the sacrum (i.e. axial skeleton), usually in a midline location [1, 3, 4, 6]. Although extremely rare, chordomas can also be located outside the axial skeleton; these are known as extra-axial chordomas (EAC) [7]. While persistent notochordal remnants are considered by many to be the origin of axial chordomas, the tumorigenesis of EAC remains unclear, as no notochordal remnants have been identified in extra-axial bone or soft tissue. Therefore, two explanations have been proposed for the mechanism by which EAC may appear; i) cellular expression of brachyury could be activated by acquired genetic or epigenetic alterations of the T-brachyury gene, driving the development of tumor cells with notochordal differentiation [8], and ii) activation in progenitor cells of a set of genes important for early embryonic development, leading to subsequent differentiation into a notochordal phenotype [9]. Although EAC have identical histological features to classic chordoma, the clinical and radiologic diagnosis can be challenging and elusive.

Many studies describing chordomas in the axial skeleton have been published [10–14]. However, studies reporting on EAC are scarce. To better understand this very rare tumor, we present a case report of a 20-year-old male with an EAC of the left distal femur, and review the current available literature regarding EAC in the lower extremities.

## Case report

A 20-year-old male, with no significant previous medical history, presented to our orthopedic outpatient clinic with a five-year history of pain in his left knee, slowly progressing over the years. The pain mainly occurred during prolonged standing. There was no history of trauma or any other event related to the knee. At time of first presentation, the patient was using analgesics (i.e. nonsteroidal anti-inflammatory drugs), which were moderately effective. The pain did not interfere with his daily activities. Physical examination showed no visible abnormalities, but limited flexion of the left knee and tenderness to palpation over the distal lateral femur was present.

### Radiology

**Fig. 1:**
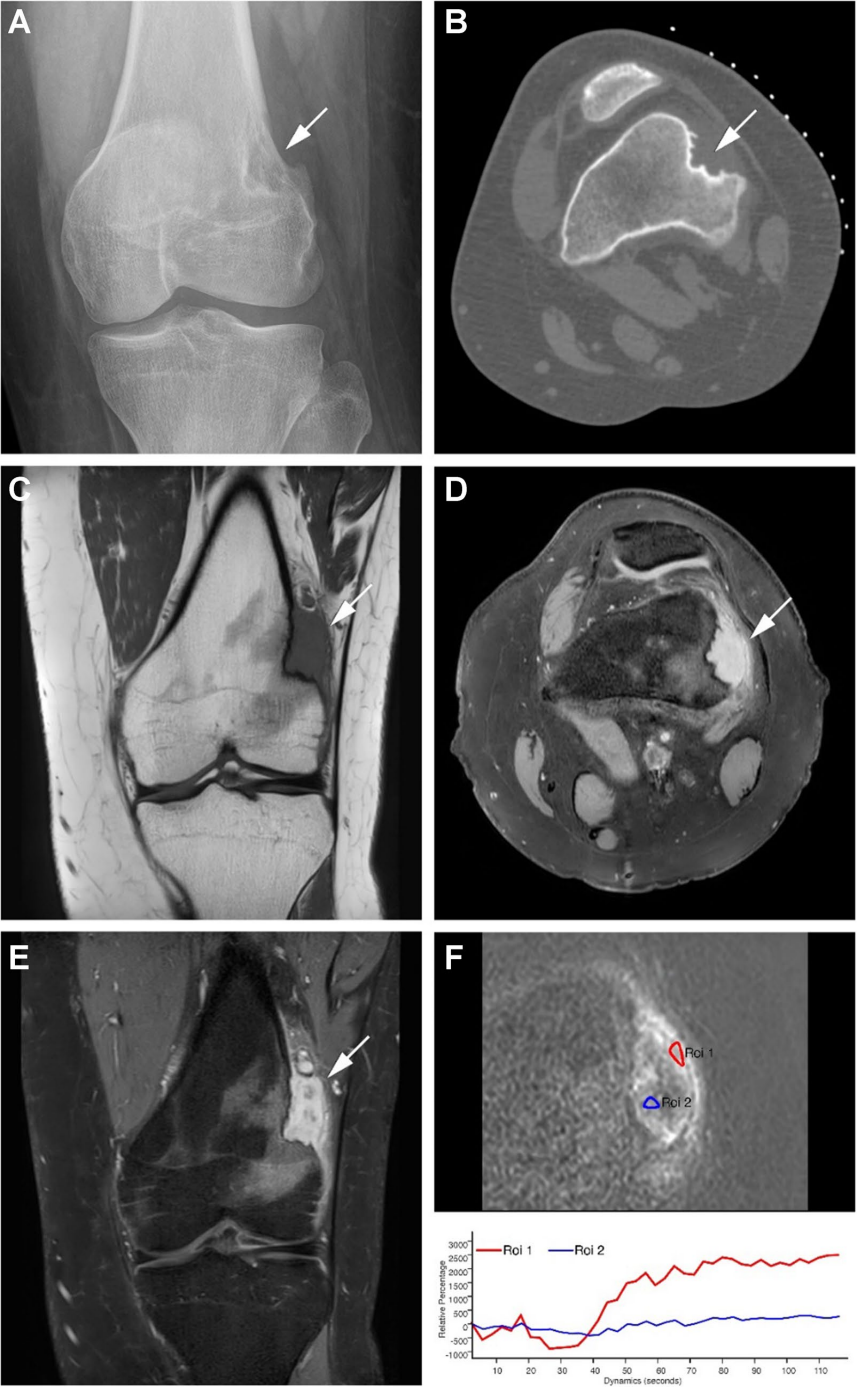
**A:** Distal femur (AP view): aggressive lytic lesion in the lateral distal femoral metaphysis eccentrically with marginal sclerosis medially but with apparent cortical destruction laterally (arrow). **Figure 1B:** CT scan (axial plane): cortical erosion with sclerotic underlying cortex (saucer-shaped defect) without matrix mineralization (arrow). **Figure 1C:** Coronal T1 weighted image (WI): lobulated and cortically based extrinsic erosion of bone and soft tissue extension (4.1 × 1.5 × 7.1 cm) in the distal femur metaphysis with an intermediate signal intensity on T1-weighted image (arrow). **Figure 1D:** Axial T2-weighted image with fat suppression (FS) and a high signal intensity: lobulated and cortically based lesion with extrinsic erosion of the distal femur metaphysis, with perifocal bone marrow edema as well as edema in the surrounding soft tissues (arrow). **Figure 1E:** Coronal T1 fat suppression (FS) contrast-enhanced weighted sequences: lesion with diffuse enhancement with small central areas lacking enhancement (arrow). **Figure 1F:** Dynamic contrast-enhanced magnetic resonance imaging (DCE-MRI) perfusion: rapid enhancement within 8 s after the artery, followed by a plateau phase; no washout was observed (type III enhancement curve) over all the tumor (RED region of interest, ROI). Several central areas in the tumor do not show contrast enhancement (type IV enhancement curve), (blue region of interest, ROI)

Radiographs revealed an osteolytic lesion with peripheral sclerosis, eccentrically located within the distal lateral metaphysis of the left femur (Fig. 1A). An additional CT scan was performed, showing cortical erosion with a sclerotic underlying cortex (saucer-shaped defect) without matrix mineralization (Fig. 1B). Magnetic resonance imaging (MRI) demonstrated a lobulated and cortically based lesion with extrinsic erosion (measuring 4.1 × 1.5 × 7.1 cm) in the distal femoral metaphysis, with iso- to slightly hyperintense signal intensity on T1-weighted images (Fig. 1C), and very high signal intensity on T2-weighted images (Fig. 1E) with extensive perifocal bone marrow edema as well as edema in the surrounding soft tissues. Contrast-enhanced T1-weighted sequences revealed diffuse enhancement with a small central areas lacking enhancement (Fig. 1D). Dynamic contrast-enhanced MRI (DCE-MRI) perfusion showed rapid enhancement within 8 s after the artery, followed by a plateau phase; no washout was observed (type III enhancement curve) in the tumor, except for several central areas that did not show contrast enhancement (type IV enhancement curve) (Fig. 1F). Consequently, an ultrasound-guided biopsy was performed to obtain tissue samples for histologic and immunohistochemical assessment.

### Histological and immunohistochemical assessment

**Fig. 2:**
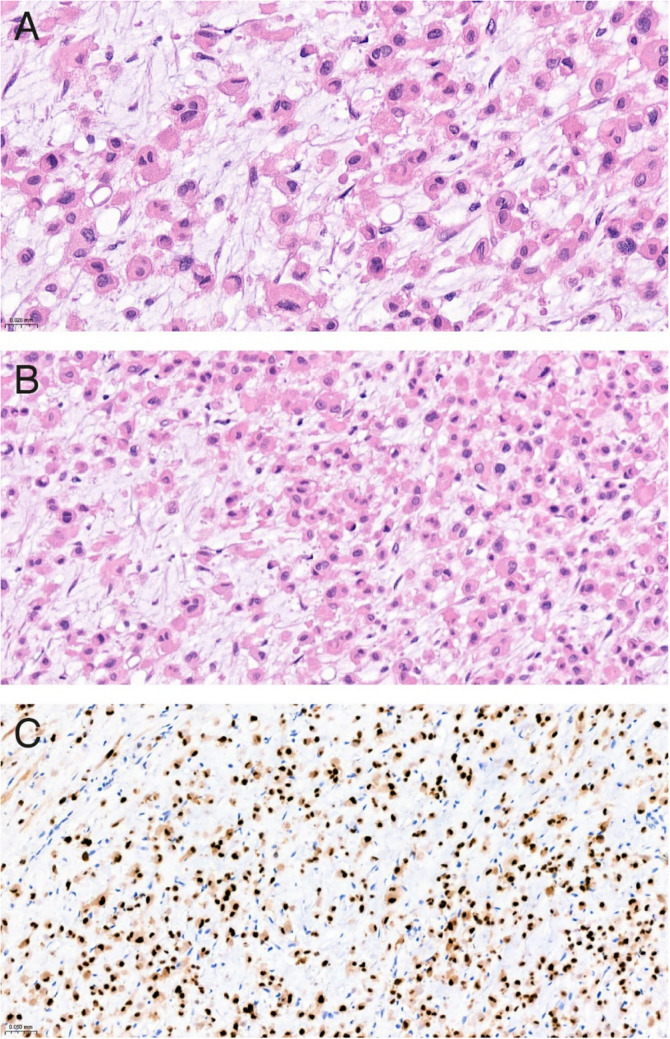
**A and 2B:** H&E staining of tumor tissue showing a moderately cellular tumor embedded in a myxoid matrix. The nuclei of tumor cells are oval to bean-shaped with variation in size and shape**. Figure 2C:** Tumor cells show strong positivity for brachyury

Biopsy revealed a moderately cellular tumor, embedded in a myxoid matrix (Fig. 2A and B). Tumor cells were scattered throughout the matrix or arranged in strands. The cells contained abundant eosinophilic cytoplasm with sharp cell borders. Nuclei were oval to bean-shaped and showed moderate atypia with variation in size and shape.

Immunohistochemically, the tumor cells were diffusely and strongly positive for brachyury and positive for Keratin AE1/AE3 and S100 staining (Fig. 2C) confirming the diagnosis of an EAC.

### Treatment and clinical course

**Fig. 3:**
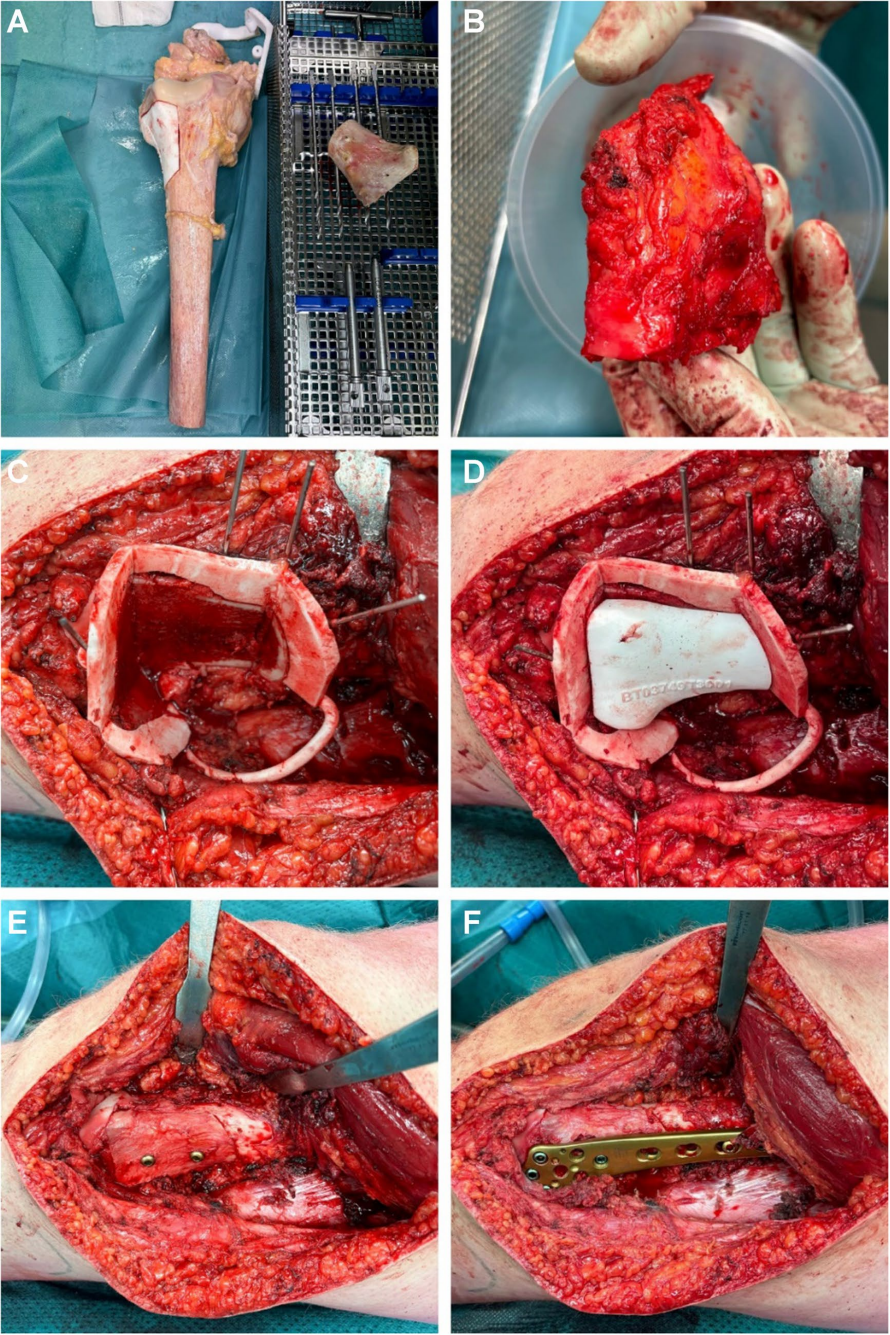
**A:** Jigsaw fitted onto the allograft bone. **Figure 3B:** Resected EAC with macroscopically free margins. **Figure 3C:** 3D-printed surgical jig fitted onto the femoral bone and fixed with K-wires. **Figure 3D:** Fitting of the jig-saw like piece of allograft. **Figure 3E:** Allograft fixated with two lag screws on the femoral bone. **Figure 3F:** Nine-hole LCP distal femur plate fixated on the femoral bone to bridge the bone defect

After the diagnosis of EAC was established, additional abdominal and thoracic CT scans showed no evidence of metastatic disease. Accordingly, an *en bloc* resection including reconstruction using an inlay allograft and a 9-hole LCP distal femur plate was performed without complications (Fig. 3).

The patient was discharged five days postoperatively and was allowed partial weight bearing (i.e. 50%) for a total duration of six weeks post-surgery.

Histological and immunohistochemical assessment confirmed the diagnosis of EAC, with free margins. During follow-up, progressive incorporation of the allograft was observed on radiographs. There are no signs of local recurrence or metastases, and the patient has returned to his daily activities at five months postoperatively.

## Discussion

Comparing the patient characteristics and presenting complaints of our patient to those described in the literature (Table 1: 13 studies describing a total of 21 adult patients) revealed many similarities. Regarding patient characteristics, the age of our patient (i.e. 20 years old) was within the range reported in the literature (i.e. 18 to 74 years, median age of 46 years). Most patients presented with pain, which was similar to the presentation of our patient. The EAC in our patient was located in the distal femur, comparable to five patients described in the literature [8, 15–18].

**Table 1:** Clinical characteristics and outcomes of adult patients with an extra-axial chordoma in either the femur/fibula/tibia

Study	Age/Sex	Location	Clinical presentation	Treatment	Follow-up and outcome
Balogh [15]	67/F	Femur (L)	Pain	Distal femoral replacement and adjuvant RTx	6-months post-surgery: alive
Huang [25]	74/M	Tibia (L)	Enlarging mass	Proximal tibia resection with megaprosthesis reconstruction	36-months post-surgery: NED
Klubícková [26]	74/M	Knee (L)	Recurrent hemarthrosis	Radical resection	8-months: NED
Kurzawa [16]	62/F	Femur (L)	Swelling and pain	Surgery (no clear margins) with proton therapy (10-months post-surgery)	28-months post-surgery: no progression
Lantos [17]	21/F	Femur (L)	Pain	Excision biopsy with bone grafting	5-years post-surgery: recurrence
Lantos [17]	21/F	Tibia (R)	Pain	Curettage - 1-year post-curettage referral to other hospital; - En bloc resection of the distal tibia, distal fibula, and the interosseous space	3-years post-surgery: NED
Lauer [27]	62/F	Popliteal Fossa (R)	-	Radical resection and adjuvant CTx	7-months: alive, with local recurrence and long metastasis
Nguyen [18]	30/F	Femur (L)	Pain	Open biopsy with curettage	4-months post-surgery: NED
O’Donell [19]	27/M	Tibia (R)	Pain	Resection	36-months post-surgery: NED
Rekhi [31]	42/M	Tibia (R)	Pain	Biopsy with curettage and bone graft	3-months post-surgery: NED
Righi [30]	67/F	Knee (unknown)	-	Two marginal resections followed by thigh amputation	43-monhts post-surgery: NED
Righi [30]	42/M	Knee (L)	-	Wide excision	22-months: NED
Sande [36]	37/M	Knee (L)	-	Marginal excision followed by transfemoral amputation (3-months). Adjuvant RTx and CTx	20-months post-surgery: DOD
Sande [36]	65/F	Knee (R)	-	Distal femur extraarticular resection with rotationplasty followed by total hip disarticulation and amputation	13-months surgery: alive, with lung metastasis
Sande [36]	63/F	Knee (L)	-	Marginal excision followed by transfemoral amputation	7-months post-surgery: alive, with lung metastasis
Sande [36]	60/F	Knee (R)	-	Marginal excision followed by transfemoral amputation	26-months post-surgery: NED
Sande [36]	68/F	Knee (L)	-	Neoadjuvant CTx. Transfemoral amputation	2-months post-surgery: NED
Tirabosco [8]	35/M	Tibia (R)	-	Resection	18-months post-diagnosis: NED
Tirabosco [8]	68/M	Femur (L)	-	Resection	40-months post-diagnosis: alive, with evidence of disease
Tirabosco [8]	18/F	Tibia (unknown)	-	Curettage	14-months post-diagnosis: alive, with evidence of disease
Wen (37)	52/F	Knee (L)	Pain and swelling	Radical resection, adjuvant CTx and RTx	21-months: alive, with metastasis

M = Male; F = Female; R = Right; L = Left; NED = No Evidence of Disease; RTx = Radiotherapy; CTx = Chemotherapy; DOD = Died Of Disease

Histological and immunohistochemical assessment are the gold standard for the definitive diagnosing of EAC. Radiologic analysis including radiographs, cross-sectional imaging with MRI, which may be supplemented with CT, are always necessary for evaluation of tumor extent, soft tissue involvement and intrinsic tissue characteristics. There are no specific radiological criteria for EAC due to their extreme rarity and overlapping features with other more common lesions such as periosteal chondroma, chondrosarcoma, and chondromyxoid fibroma. Based on our case and previous publications [15, 19], EAC of the extremities and periosteal chondroma/chondrosarcoma share some similarities on CT with cortical erosion and sclerotic underlying cortex (saucer shaped defect). Intramedullary extension can be seen both in EAC and periosteal chondrosarcoma or in chondromyxoid fibroma, but not in periosteal chondroma [20]. In addition, conventional MRI (T1 and T2-weighted images) can be misleading because both periosteal chondroma/chondrosarcoma and EAC present with very high signal intensity on T2-weighted images due to the presence of myxoid/chondromyxoid matrix. In addition, peripheral and septal enhancement is a described pattern of enhancement in chordoma and can thus appear similar to the pattern observed in chondrosarcoma [21]. Regarding chondrosarcoma, both EAC and chondrosarcoma show marked hyperintensity on T2-weighted MRI due to their high fluid content, primarily from an abundant extracellular myxoid matrix. In addition, chondrosarcoma typically demonstrate septal or nodular enhancement after gadolinium administration [21]. In contrast, chordomas may also demonstrate heterogeneous enhancement following gadolinium administration, often presenting a moderate to marked “honeycomb” pattern. Additionally, diffusion-weighted MRI with apparent diffusion coefficient mapping is useful in assessing chordomas, particularly for differentiating them from chondrosarcomas. Chondrosarcoma demonstrate the highest mean apparent diffusion coefficient values (2051 ± 261 × 10^−6^ mm^2^/s), which are significantly higher than those of conventional chordomas (1474 ± 117 × 10^−6^ mm^2^/s or lower) [22–24].

Only a few studies have systematically described the MRI findings of EAC [8, 15, 16, 19, 25–27], and to our knowledge, this is the first publication describing advanced MRI findings of this lesion using DCE. Based on a recent publication, combining morphological and functional imaging parameters is useful in making a more confident diagnosis of primary bone neoplasms in the skull base, mobile spine, and sacrum, and therefore also in axial chordomas [24]. These findings can also be extrapolated to EAC, as in our case. Therefore, the presence of curve types III and IV on DCE, combined with the other presented radiological features, increases accuracy in tumor characterization of EAC.

Histopathological characteristics of a chordoma include clusters of cells with eosinophilic to bubbly cytoplasm, located in a myxoid matrix [28, 29]. All accessible cases describing histopathological findings showed classical morphology, with epithelioid cells harboring amphophilic to eosinophilic cytoplasm embedded in a myxoid to chondromxyoid matrix [8, 15–19, 25, 26, 30, 31]. The diagnosis of EAC can be confirmed using immunohistochemical staining for brachyury (i.e. a nuclear transcription factor involved in notochord development and therefore highly specific for (extra-axial) chordomas [25, 32]). In addition, antibodies AE1/AE3 keratin are often positive in EAC [33].

The recommended treatment for patients diagnosed with EAC is surgical resection with wide margin [7]. Due to the rarity of the disease, there is limited information regarding the additional value of (neo)adjuvant radiotherapy or chemotherapy [7].

During a follow-up period ranging from two months to five years, around 40% of described cases had either local recurrence or (lung) metastasis. Due to the (relatively) high risk of both local recurrence as well as (lung and liver) metastases, it is essential to monitor patients closely to enable early detection of relapse; this includes clinical assessment, but, moreover, with radiologic imaging such as MRI, and thoracic and abdominal CT [34, 35]. The latter has been performed in six patients described in the literature [8, 19, 27].

## Conclusions

EAC in the long bones of the lower extremity is an extremely rare disease, with only 21 patients described in the literature. Histological and immunohistochemical assessment are the gold standard for the definitive diagnosing of EAC. Radiologic analysis are always necessary for evaluation of tumor extent, soft tissue involvement and intrinsic tissue characteristics. EAC may show intramedullary extension, a very high signal intensity on T2-weighted images, heterogeneous enhancement following gadolinium administration and often presents as a moderate to marked “honeycomb” pattern.

## Data Availability

Used data are accessible through the references of the included studies. Also, used data is described in Table 1.
